# Plants, Microorganisms and Their Metabolites in Supporting Asbestos Detoxification—A Biological Perspective in Asbestos Treatment

**DOI:** 10.3390/ma17071644

**Published:** 2024-04-03

**Authors:** Stanisław Łuniewski, Weronika Rogowska, Bożena Łozowicka, Piotr Iwaniuk

**Affiliations:** 1 Faculty of Economics, L.N. Gumilyov Eurasian National University, Satpayev 2, Astana 010008, Kazakhstan; astwa@astwa.pl (S.Ł.); b.lozowicka@iorpib.poznan.pl (B.Ł.); 2 Faculty of Economic Sciences, The Eastern European University of Applied Sciences in Bialystok, Ciepła 40 St., 15-472 Białystok, Poland; 3 Department of Environmental Engineering Technology and Systems, Faculty of Civil Engineering and Environmental Sciences, Białystok University of Technology, Wiejska 45E St., 15-351 Białystok, Poland; 4 Institute of Plant Protection—National Research Institute, Chełmońskiego 22 St., 15-195 Białystok, Poland; p.iwaniuk@iorpib.poznan.pl

**Keywords:** asbestos-containing wastes, naturally occurring asbestos, detoxification, biological treatment, siderophores

## Abstract

Many countries banned asbestos due to its toxicity, but considering its colossal use, especially in the 1960s and 1970s, disposing of waste containing asbestos is the current problem. Today, many asbestos disposal technologies are known, but they usually involve colossal investment and operating expenses, and the end- and by-products of these methods negatively impact the environment. This paper identifies a unique modern direction in detoxifying asbestos minerals, which involves using microorganisms and plants and their metabolites. The work comprehensively focuses on the interactions between asbestos and plants, bacteria and fungi, including lichens and, for the first time, yeast. Biological treatment is a prospect for in situ land reclamation and under industrial conditions, which can be a viable alternative to landfilling and an environmentally friendly substitute or supplement to thermal, mechanical, and chemical methods, often characterized by high cost intensity. Plant and microbial metabolism products are part of the green chemistry trend, a central strategic pillar of global industrial and environmental development.

## 1. Introduction

The definition of asbestos changes depending on the context—the term has its commercial, mineralogical, geological, regulatory, analytical, or public health and media meaning [1], but none of the most popular labels—regulatory and mineralogical—fully reflect the common understanding of the term [2]. “Asbestos” refers to the trade name of six fibrous minerals representing two groups. The first group is serpentine asbestos, known as chrysotile. The second one is amphiboles, including actinolite, amosite, anthophyllite, crocidolite, and tremolite [3,4,5,6,7].

Chemically, asbestos is a hydrated silicate containing various metals, mainly magnesium and iron. They may contain admixtures, e.g., other silicates, such as mica or talc; carbonates, such as calcite, dolomite or magnesite; and metals, including nickel, chromium, and vanadium. The composition of chrysotile is homogenous, while amphiboles’ physical properties and chemical composition are diverse. Some asbestos, e.g., crocidolite, may contain polycyclic aromatic hydrocarbons [6].

All the above minerals are strongly toxic, and their extraction, processing, and release are legislated [8]. Łuniewski and Łuniewski [9] indicated that about 150 minerals in nature in fibrous form can separate into resilient fibers (fibrils) during manufacturing. On the other hand, Baumann et al. [1] reported that roughly 400 minerals occur naturally as fibrils. Still, only the six listed above were brought to legal regulation because they were the only mineral fibers used commercially at the time of the introduction of regulations. The basis of the law was the assumption that sole commercial use could lead to widespread human exposure. Other minerals whose structures also contain fibers (e.g., erionite, taconite, and talc), although capable of causing asbestos-related diseases, are not referred to as asbestos [10].

**Figure 1:**
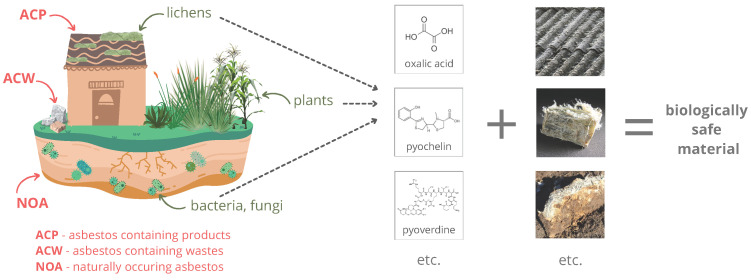
Asbestos disposal by living organisms.

Various organisms produce a wide array of compounds, including primary and secondary metabolites. Primary metabolites are necessary for normal functioning, including growth and reproduction, while secondary ones are unrequired. They are often characteristic of a particular genus or species and have enormous structural and functional variation [11]. Their production responds to external stress and is part of the interaction between the organism and the environment. The secondary metabolites can promote plant growth and protect them from insects and herbivores or drought; others may be cytotoxic inhibitory agents or characterized, for example, by antibacterial, antifungal, and antioxidant properties [12,13]. These substances can provide advantages over other species, for example, by increasing survival rates or allowing colonization of hard-to-reach areas. For instance, lichens can produce chelating and acidic compounds that enable them to colonize asbestos minerals [14] (Figure 1).

According to the policy of the WHO (World Health Organization) and the ILO (International Labour Organization), the elimination of asbestos-related diseases is possible primarily facilitated by prohibiting the use and production of all forms of asbestos, but the removal and disposal of existing and used asbestos constitutes a constant threat to human health. Many methods of asbestos detoxification are known, but they are not without environmental impacts and often involve colossal investment and operating costs. This article points out the modern direction in the disposal of this mineral using selected metabolites of microorganisms and plants. The substances can successfully dispose of naturally occurring asbestos found in industrial products, which are still present in the environment today, despite the ban on their use and marketing in many countries. Using plant and microbial metabolism products is part of green chemistry, a crucial component of the foundation in developing global industry and environmental protection. Moreover, asbestos exposure is an essential inducer of many diseases, including cancer, so the topic is also highly relevant for protecting human health and life.

## 2. Naturally Occurring Asbestos (NOA) and Asbestos Use in Industry

Asbestos is a ubiquitous mineral in the environment—its rich deposits can be found in Russia (the Urals) and the US (the Appalachians), as well as in Canada, India, China, Italy, the southern part of Africa, Greece and Cyprus [15].

Asbestos minerals can form in various rock types at a wide range of temperatures and pressures. The high magnesium content of the parent rock and suitable structural and metamorphic conditions are conducive to their formation [16]. The natural occurrence of asbestos is primarily associated with ultramafic rocks, mainly serpentinite, in which fibers are found in various generations of metamorphic veins [17]. Asbestos is also often found in mines and quarries of heavy metals or other minerals, such as chromite or vermiculite [5].

Despite this high prevalence, the belief that environmental exposure to asbestos through water or soil was negligible persisted for some time, based on the assumption that the fibers should settle on the ground and be filtered out of the water by it [18]. However, asbestos contamination of soil has an essential role in shaping human exposure to this agent, primarily when the ground is used for agricultural purposes because human activity leads to the mobilization of fibers [15]. Assuming that asbestos fibers behave similarly to other mineral colloids in water, their mobility in soil is determined by physical factors, i.e., particle size and shape, pore size distribution in the matrix, and chemical ones—pH, ionic strength, and the presence of phosphate and dissolved organic carbon, which affect the interaction between the colloid and soil. In addition, fulvic and humic acids can increase fiber mobility [19], so it can be assumed that agricultural soils rich in asbestos may be an essential factor in shaping cancer incidence.

All asbestos is of natural origin, but the term “naturally occurring asbestos (NOA)” distinguishes between fibers found in rocks and soil that can be mobilized by human activity or weathering and those that come from industry or commercial sources [16]. In addition to the natural presence of asbestos in bedrock, its presence in the environment can result from improper disposal of materials containing it or proximity to mining/treatment centers for the mineral [15]. The most commercially used asbestos mineral is chrysotile, which forms thin, soft, and flexible fibers. It accounts for about 90% of global asbestos production [8]. The properties that have led to its prevalence in many industries are primarily its mechanical strength; resistance to biodegradability; high resistance to electricity, alkalis, and acids; ability to absorb sound; poor thermal conductivity; resistance to low and high temperatures; negligible solubility in water; and non-flammability [8,20,21,22,23].

Due to its properties, asbestos had about 3000 different uses at its peak in industrialized countries [24]. One of the most popular asbestos-containing products is eternit, which has found use in the construction industry, mainly for roofing and facades. The peak of its use was in the 1960s and 1970s. The asbestos content of this product ranged from 10 to 18%, and its competitiveness was primarily due to its more attractive price compared to other products with similar characteristics [25]. In addition, different varieties of asbestos have found use in the brewing and pharmaceutical industries for filtration, as fillers for varnishes, and as insulation material for heating and reinforced wiring for various plastics.

## 3. Asbestos’ Impact on Human Health

The health effects of human exposure to asbestos have been well studied at the individual level and in larger populations, such as at the national level [26,27,28,29,30]. Asbestos use in the 1960s correlates strongly with asbestos-related disease mortality in the early 21st century. Reducing asbestos use, mainly due to appropriate regulations and restrictions, has resulted in declining disease rates. As a result, it is assumed that the most effective way to eliminate asbestos-related diseases will be to ban the usage of all asbestos forms [31]. Despite restrictions on its use and the manufacture of asbestos-containing products worldwide, annual production hovers at around 2.5 million tons, and products containing the mineral are still widely used in India and China, where more than a third of the world’s population lives [5].

The first mention of the link between asbestos and cancer incidence appeared in 1938 when the criteria for handling workplaces associated with the material were established [32]. The problem of diseases caused by asbestos minerals was for some time considered to only concern the population associated with its mining and processing, but the phenomenon affects not only workers but also their families and the population living in areas near industrial centers [4,8]. For example, car tires and clothing can carry amphiboles, exposing populations other than those directly affected by dust emissions [33]. Finally, the “ILO Resolution concerning asbestos in 2006” stated that all forms are human carcinogens [34]. The policies of the WHO and the ILO on asbestos are converging [34]; both organizations have declared combating diseases associated with the material a priority for cooperation [35]. The critical risk associated with asbestos is the release of fibers from the products, which enter the respiratory tract and cause asbestosis and carcinogenesis. Fiber lengths of 5–10 µm and diameters of 0–1 µm are considered the most dangerous [8]; by comparison, the diameter of cotton fibers is 10,000 to 35,000 nm, amphiboles are 100 to 300 nm, and chrysotile is 15 to 42 nm [9].

Asbestos has been classified as Group 1 by the IARC (International Agency for Research on Cancer), which means that there is sufficient evidence to suggest that this material is involved in causing cancer in humans [36,37,38]. Asbestos exposure justifies the formation of cancers, including malignant pleural mesothelioma [39,40,41,42,43,44,45,46], lung cancer [41,42,47,48,49,50,51], as well as ovarian cancer [52,53,54,55] and cancer of the larynx [56,57,58,59]. For cancers of the pharynx, stomach, colon, and rectum, the IARC considers asbestos an agent with limited evidence of causing the above diseases [38]. Non-malignant conditions associated with exposure to the mineral include asbestosis [1,5,47,49,51], asbestos warts, pleural effusion, pleural plaques, and diffuse pleural fibrosis [4,60]. In addition, it may be responsible for other health effects, including decreased immune function or cardiovascular disease [33]. Annual global healthcare costs associated with asbestos-related cancers hover at roughly USD 2.4–3.9 billion, excluding the charge of pain, suffering, and welfare losses [31].

## 4. Conventional Methods of Asbestos Management

Once it loses its properties or dereliction, asbestos or material containing it becomes waste, the management of which proves problematic. The most popular possibility put into practice involves landfilling. It is a temporary solution as it generates the danger of migration of finer fibers into the environment. Moreover, landfills are often not prepared to accept large amounts of waste in terms of organization and require constant supervision and high financial expenditures [61]. Moreover, such an approach is incompatible with sustainable land use, recycling, and closing material cycles [8,62]. It is necessary to dispose of asbestos, although this involves specific difficulties and costs. In addition, in many countries, despite the massive preponderance of disadvantages over advantages, landfilling is the only legally permitted method of asbestos waste disposal.

Asbestos disposal can be carried out by biological, mechanical, thermal, chemical or mechanochemical processes (Table 1). One method of asbestos disposal is solidification or stabilization [62,63,64], which involve immobilizing asbestos fibers in various matrices, such as cement or polymer resins. Their main advantages are low cost, both in terms of investment and operation, simplicity, and speed in removing the immediate danger. On the other hand, they do not eliminate fibers irreversibly, and the resulting material is not a reusable product. Another way to deal with asbestos is vitrification technology [65,66,67], which involves using high temperatures to remove organic matter and liquify the mineral fraction. The process reduces the mass and volume of the waste, and the structure and properties of the material are changed; the result of the operation is a material in the form of glass or substances similar to it, characterized by high mechanical strength and low chemical reactivity, reusable, for example, in construction. This technology does not generate additional solid waste but produces volatile pollutants. There are also huge costs: firstly, in setting up the installation and then ensuring proper system operating conditions and providing energy [68,69]. Mechanical methods are the next option for asbestos product treatment—they degrade the mineral’s crystal structure, and the fibers are destroyed by breaking the bonds between silica and brucite. The result of this approach is asbestos-free powders that can be used as raw construction material. Unfortunately, mechanical processing involves even higher costs than thermal processing [62]. Grinding can complement thermal methods—this allows a lower temperature to be used in the process and also increases the degree of amorphization of the product [70]. Chemical treatment of asbestos is another potential option for its disposal. This approach involves using organic and inorganic bases, acids, and fluorine compounds to break down the fibers. Theoretically, the end products of the process can be reused, for example, in the production of fertilizers or as an adsorbent or filler in the production of plastics [61]. It is worth noting that processes almost identical to those carried out in chemical processing occur naturally but usually on a much smaller scale. Protecting and promoting them in situ or using them under industrial conditions can result in effective asbestos disposal while respecting the environment and at a limited cost.

## 5. Living Organisms and Asbestos Disposal

Living organisms and the substances they produce are capable of destroying all sorts of materials, ranging from wood [71,72,73] and wool [74,75] to plastics [76,77,78,79], with bacteria and fungi contributing the most to these processes. Few studies are available in the literature on using microorganisms of various species and the metabolites they produce to dispose of ACW (asbestos-containing wastes) and NOA. Below, we comprehensively indicate selected interactions between living organisms and asbestos, which are particularly important for its detoxification.

### 5.1. Bacteria

Thanks to their ubiquity and metabolic activity, bacteria play an essential role in the bioremediation of many xenobiotics. It has already been shown that nitrifying bacteria, through the production of nitric acid and nitrous acid, can participate in the degradation of various types of materials, such as concrete and plaster, as well as minerals, for example, sandstone, mica, and clay. It has also been reported that these bacteria, in interaction with algae, lichens, and molds and under the influence of atmospheric factors, affect the decomposition of cement and asbestos roofing materials [80,81].

Borges et al. [82] studied the biological degradation of chrysotile and asbestos cement tiles (ACT) with the involvement of the bacteria *Acidithiobacillus thiooxidans* and filamentous fungi of the species *Aspergillus niger*. The authors also evaluated the degradation of the above materials in contact with the metabolites of the microorganisms mentioned above, that is, with gluconic acid, citric acid, oxalic acid (produced by *A. niger*), sulfuric acid (produced by *A. thiooxidans*) and water as a control. For chrysotile, this degradation was assessed by the amount of Mg released from the mineral; for the ACT, it was evaluated by considering the amounts of Mg and Ca released. Organic acids and sulfuric acid proved highly effective in promoting the degradation of chrysotile and ACT, reaching up to 80–100% of the element’s release after 30 days of experimentation, resulting in an amorphous solid when chrysotile was treated with gluconic and oxalic acids. In the degradation of asbestos-cement tiles, strong Ca release occurred first, which the authors explained by promoting the dissolution of CaCO_3_ first. Only then did the degradation of the chrysotile contained in the material occur, manifested in the release of Mg. Degradation of both materials under the influence of *A. niger* and *A. thiooxidans* was measured in submerged and solid cultures. The morphologies of chrysotile and ACT after biological treatment were similar to those observed after acid treatment, indicating the approach’s effectiveness in detoxifying asbestos fibers. Contradictory results were obtained by Mohanty et al. [83], indicating the lack of effectiveness of oxalic, malonic, and citric acids at concentrations of 100 µM in removing Fe from chrysotile. Still, the authors point out that this was most likely due to the use of low concentrations of compounds and high pH. The high pH in the presence of organic acids influences the solubilization of elements from chrysotile fibers negatively, so acids’ contribution to in situ iron extraction may be limited.

### 5.2. Fungi

Fungi can colonize virtually any environment that provides a sufficient source of nutrients and water. The colonization of the surface and species composition depends on the characteristics of that surface, primarily its permeability, moisture content, porosity, and roughness. In the case of cement, its composition also determines the variety of species able to colonize it [84]. Given the above, fungi can effectively remediate asbestos-contaminated soils—mycelium can inhibit the spread of fibers in areas where these organisms are present. Moreover, both free-living and symbiotic fungi produce secondary metabolites that can complex, precipitate or reduce elements, significantly affecting their speciation, mobility, and bioavailability, but also susceptibility to bioleaching and bioremediation of metal-rich liquid and solid matrices [85]. The metabolites they produce can modify the surface of minerals, including asbestos, for example, by increasing the solubility of iron in the structure, depriving the material of active sites involved in triggering carcinogenic mechanisms [86,87,88].

One microorganism that may play an essential role in asbestos detoxification is the filamentous fungus *Aspergillus niger*. It is a significant producer of industrially valid organic acids, including gluconic, malic, citric, and enzymes. The oxalic acid it produces is a by-product that requires removal from the line due to its toxicity and potential to form a sludge that impedes further processing. On the other hand, oxalic acid is a helpful substance in hydrometallurgical and reclamation processes [89,90,91]. This compound, being one of the factors of mineral weathering, along with the naturally occurring cycles of thawing–freezing, wetting, and drying, reduces the ability of asbestos to generate reactive oxygen species [92]. Oxalic acid is a naturally occurring organic acid of moderate toxicity and is a by-product of the chemical industry. It is possible to use this substance as a potential detoxifying substance for asbestos in situ and industrial disposal of the material [93].

### 5.3. Lichens

Lichens, obligate symbionts of fungi with cyanobacteria or green algae, play a unique role in the chemical weathering of minerals. The secretion of oxalic acid by lichen fungi is assumed to be the main factor responsible for this process in the case of asbestos since oxalate deposits are often observed at the boundary between lichen and rock. The presence of lichens on asbestos minerals leads to their chemical modification, change in physical properties, or even destruction. Physical processes, such as alternate shrinkage, surface-adherent thallus expansion, and hyphae penetration into minerals, lead to disaggregation. Lichens can release acidic and chelating molecules, leach, and/or complex metal ions, contributing to mineral dissolution or neoformation. Primary metabolites, such as oxalic acid, and a wide range of poorly soluble secondary metabolites may be involved in biogeochemical processes. Still, the effect of altering mineral properties does not occur identically for all NOAs [17].

According to Favero-Longo et al. [94], lichens of the species *Lecanora rupicola* and *Xanthoparmelia tinctina*, growing on serpentine, produce a broad spectrum of secondary metabolites (atranorin, sordidone, thiophanic acid, lecanoric acid in *L. rupicola*; salazinic acid and usnic acid in *X. tinctina*), while *Candelariella vitellina* can produce calycin and pulvinic dilactone. The production of oxalates by *L. rupicola* and *X. tinctina* on serpentinite surfaces has been observed both in the environment and under laboratory conditions. The authors report that in vitro incubation of minerals with sterile cultured isolates of lichen-forming Ascomycota only partially reproduces the natural biogeophysical and biogeochemical processes occurring at the lichen-rock interface in the field. It comes from differences in fungal metabolism, length of contact time between lichen and mineral fibers, and the lack of external dynamic factors mobilizing chemical weathering agents.

### 5.4. Yeast

There are virtually no studies on the use of yeast for asbestos detoxification. Instead, one can find information that these organisms can colonize asbestos-containing materials. Cassiola et al. [95] showed that yeast cells of the *Saccharomyces cerevisiae* species trapped in chrysotile washed with tap water and activated by sonification at pH = 4.7 to remove the brucite layer and obtain fibers of shorter length manifested fermentative activity but were less able to bud. Another study [96] evaluated the viability of *S. cerevisiae* cells on chrysotile and crocidolite activated by the method as in the aforementioned cited experiment and on chrysotile heated for three days under a reflux condenser with hot fuming hydrochloric acid to remove the brucite surface and make the fibrous layer available. The study demonstrated the effectiveness of the substrates in immobilizing cells and the fermentation activity of yeast growing adherently on the materials analyzed and stored for at least three years. However, the authors reported deformation of yeast cells by crocidolite fibers and reduced viability of microorganisms, which they attributed to the higher toxicity of this material compared to chrysotile. Wendhausen et al. [97] also reported the effectiveness of chrysotile in immobilizing yeast cells and increasing fermentation yield.

## 6. Metabolic Products of Microorganisms and Plants under Abiotic Stress Conditions

Microorganisms can adapt to adverse environmental conditions through complex, multi-level processes. Moreover, they enable plants to survive stress by promoting their growth, managing nutrients, including iron and magnesium, and fighting disease [98]. Accordingly, microorganisms can also adapt to asbestos-rich environments via physicochemical biotransformation of this mineral.

All asbestos contains relatively high amounts of iron—from 2% to approximately 27%. This element plays a structural role in asbestos or is a substitute cation or impurity [99]. Depending on the type of mineral, the amount of Fe can vary significantly [100]—in amosite, it is ~28.5%; in crocidolite, it is 27.3%; in tremolite, it is less than 5%; and in chrysotile, it is only 0.7% [101,102,103]. Fe can catalyze the Haber–Weiss (Fenton) reaction, generating OH hydroxyl radicals [100,104,105,106,107,108]. The OH-producing ability of the fibers of individual asbestos minerals is as follows: crocidolite > amosite > tremolite > anthophyllite > chrysotile. Still, a key role is played by both the Fe content and oxidation state [109]. Pacella et al. [105] found a direct correlation between Fe topochemistry and chemical reactivity. Fe^2+^ and Fe^3+^ in exposed amphibole sites are crucial determinants of Fe availability in biochemical reactions, particularly in reactive oxygen species generation. On the other hand, the relationship between crystallochemical characteristics and toxicity in vitro is more complicated. According to the above study, minerals characterized by significantly higher Fe content and chemical reactivity showed comparable toxicity to less reactive and less Fe-rich minerals. The surface reactivity of crocidolite and tremolite depends more on specific Fe sites on the outer layers of the material than on the total Fe content [104]. Fe from Mg layers loses the ability to form OH in a wide pH range caused by the precipitation of secondary Fe phases with low Fenton activity [110]. On the other hand, the complexation of Fe by ligands in the soil in the long term will not significantly reduce the generation of ⋅OH radicals by fibers due to the rapid precipitation of iron upon dissolution. Furthermore, asbestos mutagenicity and carcinogenicity may result from the interaction of Fe with ⋅NO, leading to the formation of e.g., peroxynitrites [111,112,113].

Chemical reactivity partly determines pathogenicity, including carcinogenicity, which is often linked to the presence of iron in the mineral structure [88,101,104]. Accordingly, the removal of this element, its chelation, or modification of mobilization likely represent the solution to the problem of fiber toxicity [109,114,115]. The second asbestos disposal pathway is the disruption of the magnesium-silicate bearing, which is more likely from the view of fungal than bacterial interactions [116].

### 6.1. Melanins

Melanins are metabolites produced by microorganisms that can interact with metal ions in asbestos-containing materials and thus affect their chemical and physical characteristics. These compounds are a group of dyes characterized by a high affinity for metals and the ability to bind them [86,117,118,119]. These pigments are standard in many species, e.g., fungi under stress conditions—either in the cell wall above the mannoprotein layer or extracellularly [120]. Despite their heterogeneous chemical structure, they share some common features. They have a negative charge, so they can bind metal ions by forming ionic and charge-transfer complexes. They are also resistant to temperature and acids and insoluble in most substances, including water and organic solvents [121,122]. In addition, due to the presence of quinoid groups, melanins can deactivate free radicals and peroxides and absorb electrophilic metabolites [123], which, likely, in part, enables fungi to colonize substrates where asbestos fibers are present. Elevated concentrations of toxic elements can induce or intensify the production of melanins, and their functional groups, e.g., carboxyl, amine, hydroxyl (phenolic), quinone, and semiquinone [119,124], enable the association (biosorption) of metals on the surface of microorganism cells. The various functional groups present in such pigments can contribute differently to the sorption of metals, resulting in many multiple non-equilibrium binding sites [124]. Some elements positively affect the development of chlamydospores, which are structures whose primary function is to survive unfavorable environmental conditions for an extended period. They have a high capacity for the biosorption of elements [125]. Metal binding on fungal cell wall surfaces is a passive phenomenon in living and dead cells. The process depends on the species’ ability to produce melanins or other similarly acting metabolites. Melanized cell walls tend to be thick and multilayered and thus exhibit better binding capacity than thinner cell walls. Other factors affecting metal binding efficiency include the concentration of microorganisms, the radius of ions to be bound, and the pH of the environment [126]. Asbestos weathers more strongly in an acidic environment [108]. On the other hand, such a reaction negatively affects ion binding in fungal cell walls [126].

Some common reactions were found to occur in fungi in contact with asbestos fibers. Among these, the authors singled out pigment production, the intensity of which depended on the species of microorganism. This compound may also be indirectly involved in the mechanical degradation of minerals, including asbestos. With the progressive leaching and binding of iron from the material’s structure, the surface’s porosity increases, allowing water to penetrate deeper and maintain biochemical processes at greater depth [87,127].

### 6.2. Siderophores

Although asbestos minerals are highly durable, atmospheric factors strongly affect the removal of elements from the mineral structure, determining changes in surface reactivity and crystal structure while maintaining fibrous forms [92]. Removal of iron from asbestos materials is possible, among other things, with the participation of microorganisms capable of producing siderophores—low-molecular-weight compounds that chelate iron ions. These are secondary metabolites (molecular weight < 1500 Da) produced by bacteria, fungi, and grass plants that promote the absorption of this trace element. Low intracellular concentrations of iron, linked to its bioavailability in the nutrient solution, induce their production. Siderophores provide a competitive growth advantage under conditions where the total concentration of iron in the medium is saturated, but the proportion of the bioavailable form is low [128]. These compounds contribute highly to the mobilization of metals in the environment but also enable their detoxification, preventing the formation of cellular oxidative stress [129]. In addition, they show greater efficiency in binding Fe(III) in soil compared to low-molecular-weight organic acids [130].

In a study by Bhattacharya et al. [114], bacteria from the *Bacillus subtilis* and *Bacillus atrophaeus* species showed a 40–70% reduction in asbestos-containing iron, likely through the production of siderophores. Biosynthesis of siderophores in bacteria occurs through nonribosomal peptide synthetase (NRPS) enzymes, polyketide synthase (PKS) enzymes, and/or NRPS-independent siderophore (NIS) synthetase enzymes [131]. A high capacity for scavenging iron from minerals through the release of chelating molecules, including but not limited to siderophores and some organic acids, has also been demonstrated by soil fungi of the *Fusarium oxysporum* species [132], as well as *Aspergillus tubingenesis* and *Coemansia reversa* [133], *Aspergillus fumigatus*, *Cladosporium cladosporioides*, *Verticillium lecanii*, *Penicillium chrysogenum*, *Trichoderma harzianum* and *Aerobasidium pullulans* [134]. This activity modifies the asbestos crystalline and chemical structure, reducing the reactivity of its fibers and even their deactivation. Daghino et al. [132] report that chrysotile, crocidolite, and amosite do not inhibit the growth of *F. oxysporum* soil fungi under laboratory conditions. According to Daghino et al. [135], *Verticillium leptobactrum* fungi have a high ability to extract Fe from chrysotile fibers but also effectively remove Mg and Si, which contributes to structural changes and reduced durability of the mineral. In the above study, unlike *F. oxysporum*, *V. leptobactrum* did not accumulate silicon released from the fibers. An essential role in the degradation of chrysotile fibers is the dissolution of FeIII and probably AlIII. This process leads to the labilization of Si layers, which may positively affect the rate of Si dissolution. Siderophores show potential effectiveness in dissolving chrysotile in neutral soils. Weaker biogenic ligands, e.g., oxalate, show low efficiency in degrading asbestos fibers because they cannot induce Si labilization in this environment. It is worth noting that the rate of weathering of asbestos minerals in the soil is highly dependent on the properties of the soil, especially the pH—asbestos weathering is faster at acidic pH; materials made from asbestos and cement weather much more slowly. Cement plays a significant role in changing the kinetics of this process, which is explained by two phenomena—its presence increases the pH of the soil solution, and there is also a precipitation of Al, which forms a shield on the surface, so to speak, protecting the material from dissolution [108,110]. Mohanty et al. [83] also point to the effectiveness of siderophores in the bioremediation of asbestos. According to the authors, bacterial (Desferrioxamine B) and fungal (Iron-free Ferrichrome) siderophores were equally effective in removing iron from chrysotile fibers. However, the fungal counterpart was more effective in reducing reactive oxygen species, i.e., potentially decreasing the material’s toxicity more efficiently. Desferrioxamine B is produced by the bacteria *Streptomyces pilosus* [136], and its effectiveness in increasing the solubility of iron contained in hornblende [130] and kaolinite [137] has already been described.

Bacteria of the genus *Pseudomonas* can use asbestos cement as a source of iron and magnesium. The microorganisms can produce two types of siderophores—pyoverdine (PVD), a group of green-fluorescent compounds, and pyochelin (PCH). PVDs are a group of green-fluorescent compounds that are synthesized under iron-deficient conditions. They consist of three parts: (i) a conserved fluorescent dihydroxyquinoline chromophore; (ii) an acyl side chain (either dicarboxylic acid or amide) bound to the amino group of the chromophore; and (iii) a variable peptide chain linked by an amide group bound to the C1 (rarely C3) carboxyl group of the chromophore, with the composition and length of the peptide unique to specific strains. They are involved in the iron uptake system in fluorescent pseudomonads [138,139,140,141,142]. PCH, along with enantio-pyochelin, due to their lower affinity for Fe compared to PVD, is referred to as secondary siderophores in *Pseudomonas*. Both compounds are condensation products of salicylate and two cysteine molecules, which undergo cyclization when they combine and undergo some modifications. The only difference between them is the stereochemical configuration of the two incorporated cysteines [143,144]. Both PVD and PCH participate in the dissolution of Fe from asbestos waste; the exclusion of either significantly reduces the element removal from the material. In addition, bacterial contact with the waste resulted in the repression of siderophore biosynthetic pathways, indicating the presence of bioavailable iron [136,145]. Removal of Fe from other asbestos materials by PVD has also proven effective, but the efficiency of the process depends on the type of waste and PVD. For example, PVD with *Pseudomonas mandelii* is more efficient in extracting Fe from chrysotile gypsum, while PVD with *Pseudomonas fluorescens* shows greater efficiency for amosite gypsum [80]. Fe^3+^ is bound to PCH with a 2:1 stoichiometry (PCH to Fe^3+^), where one PCH molecule is coordinated tetradentately with Fe^3+^ and the other is bound bidentately to complete the hexacoordinate octahedral geometry [146,147,148,149,150]. PVD has a stronger affinity for Fe^3+^ and forms complexes with it in a 1:1 ratio [151,152,153,154]. PVD and PCH are more effective in extracting iron from asbestos fibers than EDTA and the supernatants analyzed by the authors with the compounds mentioned above are more effective than *Pseudomonas* bacteria. Contact of the bacteria with asbestos fibers provided them with an iron source and resulted in the coating of the material with biofilm [155].

### 6.3. Phytosiderophores

Phytosiderophores are a group of chelating ligands produced by grass crops, e.g., wheat, barley, rye, oats, and corn. These plants are among the most popular cereals grown worldwide due to their use in the production of food and feed products. These compounds may also be important in the in situ degradation of asbestos due to their ability to mobilize Fe and other metals [110]. Among the elements competing with Fe for complexation by the ligand are Cu, Ni, Zn, Co, and Mn [107,156], i.e., elements that are macro- and micronutrients naturally occurring in the soil in relatively large quantities [157,158,159]. Microbial and plant siderophores differ structurally—in siderophores produced by microorganisms, hydroxamic and catechol ligand donors are present, while those produced by plants use carboxyl, amino, and hydroxyl groups as iron ligands. Phytosiderophores include mugineic acid, avenic acid, and distichonic acid, among others [160]. Plants find it easiest to accumulate elements in the form of free ions, and iron contained in serpentinites is difficult for them to access. Mugineic acids are synthesized in plant roots and secreted into the rhizosphere. Afterward, the difficult-to-solubilize FeII is solubilized through chelation. Next, the roots uptake the complexes, thus affecting the crop’s growth and quality. These compounds provide sufficient iron, which is very poorly soluble at relatively high pH and at high concentrations of Ca^2+^ and Mg^2+^ ions, hindering Fe uptake [161,162].

Phytosiderophore activity, combined with topsoil stabilization and minimization of erosion by plants, which can reduce airborne asbestos exposure, seems a promising direction for in situ asbestos disposal. Herbaceous plants of the species *Cymbopogon citratus* and *Chrysopogon zizanioides* are successful in the phytoremediation of chromite-asbestos mine waste. This type of waste is challenging for plants to colonize because of its low water-holding capacity, relative homogeneity, low bulk density, sand texture and poor nutrient content, and high chromium and nickel contents. Using plants demobilizes asbestos fibers, makes it possible to inhibit soil erosion, and improves the environment’s aesthetic value [163]. Effectiveness in phytoremediation of chromite-asbestos waste, manifested by metal accumulation, is also demonstrated by a plant cover consisting of *Cynodon dactylon, Silene nutans, Acacia concinnia*, and *Cajanus cajan* [164]. Mosses, also belonging to the plant kingdom, significantly contribute to global biogeochemical cycles and can effectively reduce the mobility of asbestos fibers. Asbestos fibers get caught up in the moss growing on the covering. Moreover, the presence of mosses accelerates the weathering of the material; on the other hand, mosses deposited on old roof surfaces can prevent the release of fibers, so they should not be removed [165].

Phytostabilization may be a viable strategy for remediating asbestos-contaminated sites, such as post-industrial sites, which are currently untreated due to the prohibitive cost of other technologies. Among the advantages of this approach, the greening of abandoned sites, the stabilization of topsoil, which reduces airborne dispersion of fibers, and the increase in soil fertility following the application of additives with high metal-binding capacity are cited. On the other hand, although asbestos itself does not affect plant health, insufficient nutrients or high levels of other contaminants can be harmful, so it is essential to consider other soil parameters as well, and not just the contamination of the site with asbestos or associated metals [5].

## 7. Conclusions

This article discusses the use of microorganisms and plants in asbestos detoxification. We suggest focusing more attention on microbial metabolites and phytoremediation.

Biological processes are useful in asbestos detoxification and their effectiveness ranges from 40% to nearly 100%. However, to obtain satisfactory results, their effect must be multiplied, which can be achieved by combining the action of microbial metabolites with other substances and factors. Of particular interest is oxalic acid, a widespread product of microbial metabolism, mainly due to its easy availability, production, and low cost. In addition, it is worth noting other low-molecular organic acids, including acetic, citric, lactic, or malonic; and mineral acids, including hydrochloric, nitric, nitrous, sulfuric, carbonic, and phosphoric. Despite the lower efficiency of biological processes, these approaches have significant advantages over thermal, mechanical and chemical methods in terms of cost (e.g., for providing energy for high-temperature or grinding processes, the cost of chemical treatments associated with the need to use large quantities of hazardous substances, their storage, treatment of the final product) and environmental impact. In view of the above, the most optimal way to dispose of ACW therefore seems to be a combination of the use of microbial metabolites and conventional methods, which would potentially reduce the environmental impacts and process costs by reducing energy consumption for mechanical and thermal processes/reagents for chemical processes, while obtaining safe, inactive material. In turn, biological methods can be successfully used for NOA detoxification. Further research in this area should focus primarily on creating combinations of biological and conventional processes, optimizing them and defining their end products.

## Figures and Tables

**Table 1:** The most important features of asbestos processing methods.

	Biological Treatment	Mechanical Treatment	Thermal Treatment	Chemical Treatment	Mechanochemical Treatment
Pre-treatment	Usually no	No	Usually yes—e.g., crushing	Usually yes—to obtain an appreciable conversion of the asbestos fibers	Usually no
Process temperature (°C)	20	n.a.	650–1600	25–600	80
Energy consumption (kWh/kg)	-	>1.5	0.5–1.5	Nonsignificant	n.a.
Chemicals consumption	No	No	No	Yes	No
Gaseous output	No	Yes—with significant amounts of dust	Yes—with small amounts of dust	No	Yes—with small amounts of dust

## Data Availability

No new data were created or analyzed in this study. Data sharing is not applicable to this article.
